# Childhood asthma, asthma severity indicators, and related conditions along an urban-rural gradient: a cross-sectional study

**DOI:** 10.1186/s12890-016-0355-5

**Published:** 2017-01-05

**Authors:** Joshua A. Lawson, Donna C. Rennie, Don W. Cockcroft, Roland Dyck, Anna Afanasieva, Oluwafemi Oluwole, Jinnat Afsana

**Affiliations:** 1 Department of Medicine and Canadian Centre for Health and Safety in Agriculture, University of Saskatchewan, 104 Clinic Place, PO Box 23, Saskatoon, SK S7N 5E5 Canada; 2 College of Nursing and Canadian Centre for Health and Safety in Agriculture, University of Saskatchewan, Saskatoon, SK Canada; 3 Department of Medicine, University of Saskatchewan, Saskatoon, SK Canada; 4 Canadian Centre for Health and Safety in Agriculture, University of Saskatchewan, Saskatoon, SK Canada

**Keywords:** Asthma, Wheeze, Urban, Rural, Children, Morbidity, Prevalence, Asthma-like symptoms, Allergic conditions

## Abstract

**Background:**

Asthma prevalence is generally lower in rural locations with some indication of an urban-rural gradient. However, among children with asthma, certain rural exposures thought to protect against the development of asthma could aggravate the condition. We examined childhood asthma prevalence and related conditions along an urban-rural gradient and also examined the characteristics of those with asthma along the urban-rural gradient.

**Methods:**

In 2013 we completed a cross-sectional survey of 3509 children aged 5–14 years living in various population densities of Saskatchewan, Canada. Location of dwelling was identified as belonging to one of the following population densities: large urban region (approximately 200,000), small urban (approximately 35,000), or rural (small town of <1,500 or farm dweller). Physician-diagnosed asthma and asthma-related symptoms were ascertained from responses in the parental-completed questionnaires.

**Results:**

Of the study population, 69% lived in a large urban region, 11% lived in a small urban centre and 20% were rural dwellers. Overall, asthma prevalence was 19.6% with differences in asthma prevalence with differences between locations (large urban = 20.7%; small urban = 21.5%; rural = 15.1%; *p* = 0.003). After adjustment for potential confounders, the association between location of dwelling and asthma remained significant. Despite a lower prevalence of asthma in the rural area, the prevalence and risk of ever wheeze and having more than 3 wheezing episodes in the past 12 months among those who reported asthma, was higher in rural locations after adjustment for potential confounders.

**Conclusions:**

The results of this study support the evidence of a difference in childhood asthma prevalence between urban and rural locations and that once a child has asthma, certain rural exposures may aggravate the disease.

## Background

Asthma is one of the most common diseases among children; but its prevalence varies geographically. Rural and farming locations have been the focus of investigation in relation to childhood asthma [1–6] as exposures thought to be linked to asthma have been shown to vary between these and urban locations [7–11]. Furthermore, access to health care is different between urban and rural populations, which might explain variations in disease prevalence.

Results from previous work suggest that asthma prevalence is lower in rural or farming locations [1, 3] although these results are not consistent [12–14]. Some of this inconsistency may result from a lack of variability in exposures within a region. For example, when making town to farm comparisons, the exposures between the two can be similar. Thus, it can be advantageous to consider multiple exposure categories by rurality. There is limited work investigating asthma along an urban-rural gradient among children and adolescents [3, 15–17]. Limitations in the previous work include secondary analyses of datasets and restricted ability to account for important confounders making it difficult to assess the true relationship of asthma across an urban-rural gradient.

To add complexity, while some exposures thought to protect against the development of asthma (e.g. endotoxin), are higher in rural areas [9, 18, 19], there is evidence that these exposures may exacerbate asthma among those with asthma [20, 21]. There is also evidence that asthma in rural dwelling children may not be under optimal control [22, 23] and may be underdiagnosed for asthma [22]. For these reasons, it is important that we also consider asthma morbidity along the urban-rural gradient.

Our overall objective was to examine asthma and asthma indicators in schoolchildren along an urban-rural gradient. We specifically wished to (1) determine if these indicators differed along an urban-rural gradient and (2) determine if asthma morbidity differed by location of dwelling among children with asthma.

## Methods

### Study design and location

The study used a cross-sectional survey design. Children were recruited from across an urban-rural gradient representing differences in population size and density in the province of Saskatchewan, Canada in spring 2013. These areas were chosen based on their population size and density as well as the lack of previous asthma research in these areas. It included large urban (Regina: population approximately 200,000), small urban (Prince Albert: population approximately 35,000), and rural (towns around Prince Albert: <2,500 people or living on a farm in the region around Prince Albert) areas. The urban-rural gradient chosen here parallels Statistics Canada definitions based on modified Beale codes where our definitions of large urban, small urban, and rural match those of small metropolitan (urban settlements of 50,000 to 249,999 people), non-metropolitan small city zone (20,000–49,999 people) and rural (<2,500 people) [24].

### Study population, selection, and recruitment

Initially, meetings were held with the school division directors followed by communication with school principals. Schools in the large urban centre were randomly selected due to the large number of schools. All schools in the Prince Albert region and the surrounding areas under the same school division administration were selected for small urban and rural settings. Once a school was selected, all children from Kindergarten to Grade 8 (approximately 5–14 years) were eligible to participate. Study packages, including an information letter and survey, were distributed to students who then took the survey home for self-completion by parents. Surveys were then returned to the school where they were collected by research staff. The total sample size was 3,509. The participation rate was approximately 28% overall (large urban = 26%; small urban = 23%; rural = 38%).

This study was approved by the University of Saskatchewan Biomedical Research Ethics Board. Parents completed the survey, and thus completion and return of the survey implied consent. All school divisions involved approved the study.

### Survey data collection and operational definitions

Surveys were based on standardized questionnaires including the International Study of Allergy and Asthma in Childhood questionnaire [25], American Thoracic Society Children’s Respiratory Disease questionnaire [26], and questionnaires used previously in Saskatchewan lung studies [19, 27, 28]. Information was collected on lung and general health, indoor environment, health behaviours, and socio-demographics.

Our independent variable of interest was location of dwelling. This was based on the classification of large urban, small urban, and rural as defined above.

Ever asthma was defined from the question: “Has this child ever been diagnosed as having asthma by a doctor?” Those with a positive response to ever asthma as well as a report of a positive response to any of the following in the past 12 months: wheeze, health care utilization for asthma (i.e. doctor visit, emergency visit, or hospitalization), asthma episodes, or breathing medication use were classified as having current asthma. Ever wheeze was defined as a positive response to the question: “Has this child ever had wheeze or whistling in the chest in the past?” A positive response to ever wheeze along with a positive response to “Has this child had wheezing or whistling in the chest in the past 12 months?” indicated current wheeze.

Asthma severity indicators (wheeze frequency, number of physicians’ visits, and asthma medication use) were also considered. Wheeze frequency was based on the question “How many attacks of wheezing has this child had in the past 12 months (0, 1–3, >3)?” Wheeze with shortness of breath was based on the question “In the past 12 months, has wheezing ever been severe enough to limit your child’s speech to only one or two words at a time between breaths?” (Present/absent). Number of physician visits for asthma was based on the question “In the past 12 months, how many times has this child required health care for asthma from the following places (Doctor’s office, 0, 1, >1)”. Breathing medication use was based on “In the past 12 months, has this child taken medicine that your doctor prescribed for a breathing problem?”

Potential confounder information was obtained from the survey and based on previous literature. These included age, sex, mother’s education level, ethnic background, mother’s smoking status, father’s smoking status, home dampness, and home mould.

### Statistical analysis

All analyses were completed using the Statistical Package for the Social Sciences (SPSS) version 23 (IBM Corporation). Descriptive statistics using means (standard deviations) for continuous data and frequencies (%) for categorical data were calculated. Prevalence of the outcomes of interest were calculated and reported by location of dwelling. Statistical comparisons were made using the independent samples chi-squared test.

Multiple logistic regression was used to adjust for potential confounders with the strength of association based on the odds ratio (OR) and 95% confidence intervals (CI). The primary independent variable was location of dwelling. Binary multiple logistic regression and multinomial multiple logistic regression were used when the outcome had two categories and more than two categories, respectively. Potential confounders listed above were included in each model. Models were first fitted for the full population then repeated including only those children with a report of asthma. An alpha level of <0.05 defined statistical significance. In a sub-analysis, we compared the rural non-farm and rural farm dwelling populations to ensure comparability.

## Results

The regions differed on most socio-demographic indicators considered (Table 1). Compared to those in the small urban area, children from the large urban centre had a higher proportion of mothers with greater than high school education and were more likely to be Caucasian. The large urban centre had a lower proportion of children with a mother or father who smoked or had lived in homes with dampness or mould than those in the small urban centre. When comparing large urban to rural children, rural children were more likely to be Caucasian or have mothers or fathers who smoked. Rural children were also about 5 months older on average than large urban children. Finally, compared to small urban children, rural children were more likely to be Caucasian but less likely to have a mother or father who smoked or live in homes with dampness or mould.

**Table 1:** Socio-demographic characteristics of the study population by location of dwelling

	Overall	Location of dwelling			
	(*n* = 3,509)	Large Urban (*n* = 2380)	Small Urban (*n* = 415)	Rural (*n* = 714)	*p*-value
Median age (25th percentile, 75th percentile)	9.0 (7.0–11.0)	9.0 (7.0–11.0)*†	10.0 (7.0–12.0)‡	10.0 (7.38–10.0)	0.004
% Female	50.5	50.9	54.1‡	47.0	0.053
% >high school (maternal)	76.2	78.8*	61.6‡	75.7	<0.001
Ethnic background					
% Caucasian	64.1	64.8*†	39.7‡	76.1	<0.001
% First Nations or Metis	15.8	8.8	50.5	18.9	
% Other ethnic background	20.1	26.4	9.9	5.0	
% Maternal smoking	17.3	13.1*†	39.2‡	18.8	<0.001
% Paternal smoking	23.3	19.0*†	45.9‡	25.5	<0.001
% Dampness in the home	16.2	14.9*	22.7‡	17.4	0.001
% Home mould	11.9	11.0*	19.2‡	10.9	<0.001

*p < 0.05 comparing large urban to small urban

†*p* < 0.05 comparing large urban to rural

‡*p* < 0.05 comparing small urban to rural

The overall prevalence of ever asthma was 19.6%. This differed by location of dwelling (Table 2) with the highest prevalence in small urban children (21.5%) and the lowest prevalence in rural children (15.1%). Similar patterns were seen for current asthma and current cough (Table 2). There was a statistically significant linear trend where the prevalence of wheeze limiting speech increased from large urban to rural.

**Table 2:** Prevalence of lung conditions by location of dwelling

	Overall	Location of dwelling				
	(*n* = 3,509)	Large Urban (*n* = 2380)	Small Urban (*n* = 415)	Rural (*n* = 714)	*p*-value overall	*p*-value (trend)
% with ever asthma	19.6	20.7†	21.5‡	15.1	0.003	0.003
% with current asthma	14.1	14.9†	15.7‡	10.9	0.03	0.02
% with ever wheeze	27.8	27.2	31.8	27.4	0.16	0.61
% with current wheeze	13.7	14.0	13.4	13.1	0.83	0.55
% with report of respiratory allergy	29.6	30.6	27.0	27.6	0.15	0.08
% with report of hay fever	4.9	4.8	4.6	5.5	0.75	0.56
% with report of eczema	17.2	17.4	15.7	17.1	0.68	0.69
% with current cough	21.3	22.3†	21.8	17.9	0.04	0.02
% with cough disturbing sleep	30.3	30.7	29.1	29.8	0.76	0.56
% with wheeze limiting speech	7.4	6.3†	8.1	11.2	0.10	0.03
% with wheeze with exercise	9.4	9.3	11.6	8.5	0.23	0.83
% taking breathing medications in the past 12 months	15.3	15.7	16.0	13.6	0.36	0.22

*p < 0.05 comparing large urban to small urban

†*p* < 0.05 comparing large urban to rural

‡*p* < 0.05 comparing small urban to rural

After adjustment for potential confounders, statistically significant inverse associations were observed for ever asthma, current asthma, current cough, and breathing medication use in the past 12 months comparing rural to large urban children, each with dose response relationships and a significant trend (Table 3). While there was not a statistically significant association between a specific location of dwelling and current wheeze, the trend across the location of dwelling gradient was statistically significant (*p* = 0.04).

**Table 3:** Adjusted^a^ associations between location of dwelling with asthma and asthma-like symptoms

	Large urban (reference)	Small urban OR (95%CI)	Rural OR (95%CI)	*P*-value for trend
Ever asthma	1.00	0.74 (0.51–1.07)	0.57 (0.44–0.75)^†^	<0.001
Current asthma	1.00	0.84 (0.56–1.28)	0.58 (0.42–0.99)^†^	0.001
Ever wheeze	1.00	0.99 (0.72–1.36)	0.84 (0.68–1.05)	0.15
Current wheeze	1.00	0.72 (0.47–1.11)	0.76 (0.57–1.01)^‡^	0.04
Respiratory allergy	1.00	0.86 (0.63–1.17)	0.83 (0.67–1.03)^‡^	0.08
Hay fever	1.00	0.86 (0.41–1.79)	1.20 (0.77–1.86)	0.48
Eczema	1.00	0.88 (0.61–1.28)	0.83 (0.64–1.07)	0.15
Current cough	1.00	0.79 (0.56–1.11)	0.75 (0.59–0.96)^†^	0.02
Wheeze limiting speech	1.00	0.96 (0.34–2.71)	1.55 (0.79–3.05)	0.23
Wheeze with exercise	1.00	1.06 (0.67–1.68)	0.80 (0.56–1.12)	0.23
Breathing medication use in the past 12 months	1.00	0.86 (0.59–1.27)	0.72 (0.55–0.96)^†^	0.02

^†^ *p* < 0.05 compared to the reference group

^‡^ *p* < 0.10 compared to the reference group

^a^Adjusted for age, sex, mother’s education level, ethnic background, mother’s smoking status, father’s smoking status, home dampness, home mould

Among children with ever asthma, there was an increasing prevalence across the urban-rural gradient observed for reporting ever wheeze, wheeze limiting speech, wheeze with exercise, more than 3 episodes of wheeze in the past 12 months, and nocturnal symptoms (Table 4). Differences were also found between dwelling locations for the number of physician visits for asthma in the past year and missing school because of breathing problems (Table 4).

**Table 4:** Respiratory symptoms and morbidity indicators by location of residence among those with ever asthma

	Overall	Location of dwelling				
	(*n* = 669)	Large Urban (*n* = 480)	Small Urban (*n* = 84)	Rural (*n* = 105)	*p*-value	*p*-value (trend)
% with ever wheeze	79.8	76.6	85.5	90.1	0.003	0.001
% with current wheeze	48.4	46.3	51.9	55.1	0.23	0.09
% with report of respiratory allergy	60.5	59.8	54.8	68.6	0.13	0.20
% with report of hay fever	12.0	11.3	8.3	18.1	0.08	0.12
% with report of eczema	32.4	32.3	23.8	40.0	0.06	0.34
% with current cough	46.8	45.2	53.6	48.6	0.34	0.33
% with wheeze limiting speech	13.0	10.9	15.3	19.2	0.13	0.04
% with wheeze with exercise	36.5	34.2	40.0	44.2	0.12	0.04
% taking breathing medications in the past 12 months	56.9	55.0	60.8	62.5	0.29	0.12
% with >3 episodes of wheeze in the past 12 months	14.5	12.3	14.3	24.8	0.004	0.002
% with nocturnal symptoms	53.8	50.4	61.9	62.9	0.02	0.01
Physician visits for asthma						
% with no visits	56.7	54.6	65.5	59.0	0.001	0.89
% with 1 visit	20.8	24.8	7.8	13.3		
% with >1 visit	22.6	20.6	27.4	27.6		
% with school missed because of breathing problems	50.6	47.4	62.5	55.9	0.02	0.03

After adjustment for potential confounders, among children with asthma, rural dwelling children were at increased risk of ever wheeze and having more than 3 episodes of wheeze in the past 12 months compared to large urban children (Table 5). A statistically significant (*p* < 0.05) trend in the association between the location of dwelling and the number of physician visits for asthma in the past year was also observed. There were borderline statistically significant inverse associations between living in small urban and rural locations with children having 1 physician visit (*p* < 0.10) compared to large urban dwelling but no such association seen for having more than 1 physician visit for asthma. Borderline statistically significant associations (*p* < 0.10) were also observed for increased risk of wheeze after exercise and missing school due to breathing problems among rural compared to large urban dwellers.

**Table 5:** Adjusted^a^ associations between location of dwelling with respiratory symptoms and indicators of asthma morbidity among those with ever asthma

	Large urban (reference)	Small urban OR (95%CI)	Rural OR (95%CI)	*P*-value for trend
Ever wheeze	1.00	2.49 (0.87–7.10)^‡^	2.93 (1.25–6.86)^†^	0.007
Current wheeze	1.00	0.99 (0.49–1.99)	1.25 (0.74–2.10)	0.44
Respiratory allergy	1.00	0.98 (0.48–2.00)	1.29 (0.76–2.19)	0.38
Hay fever	1.00	0.36 (0.08–1.64)	1.72 (0.87–3.41)	0.20
Eczema	1.00	0.77 (0.37–1.60)	1.24 (0.74–2.08)	0.52
Current cough	1.00	1.28 (0.64–2.58)	1.18 (0.71–1.98)	0.46
Wheeze limiting speech	1.00	1.09 (0.32–3.56)	1.79 (0.79–4.04)	0.17
Wheeze with exercise	1.00	1.43 (0.71–2.88)	1.60 (0.97–2.64)^‡^	0.06
Breathing medications in the past 12 months	1.00	1.66 (0.80–3.47)	1.31 (0.77–2.23)	0.23
>3 episodes of wheeze in the past 12 months	1.00	0.85 (0.29–2.44)	2.40 (1.29–4.47)^†^	0.009
Nocturnal symptoms	1.00	1.98 (0.96–4.09)^‡^	1.44 (0.86–2.42)	0.09
School missed because of breathing problems	1.00	1.59 (0.76–3.35)	1.56 (0.93–2.63)^‡^	0.07
Physician visits for asthma in the past year (ref: none)				
1 visit	1.00	0.35 (0.11–1.09)^‡^	0.49 (0.24–1.03)^‡^	0.03
>1 visit	1.00	0.66 (0.29–1.51)	0.92 (0.50–1.71)	0.68

^†^ *p* < 0.05 compared to the reference group

^‡^ *p* < 0.10 compared to the reference group

^a^Adjusted for age, sex, mother’s education level, ethnic background, mother’s smoking status, father’s smoking status, home dampness, home mould

In a sub-analysis, we split our rural group into rural non-farm dwelling and rural farm dwelling to compare these two categories of rural children. Many of the demographic and environmental characteristics as well as respiratory symptoms did not differ significantly between the two groups (Table 6). However, rural farm dwelling children had a statistically significant higher proportion of mothers with greater than a high school education (78.1%) compared to rural non-farm dwellers (68.9%).

**Table 6:** Comparison of rural non-farm and rural farm populations

	Rural non-farm (*n* = 191)	Rural Farm (*n* = 523)	*p*-value
*All Children*			
Mean age (SD), years	9.8 (2.6)	9.5 (2.6)	0.26
% Female	43.1	48.4	0.21
% >high school (maternal)	68.9	78.1	0.01
Ethnic background			
% Caucasian	70.1	78.3	
% First Nations or Metis	24.1	17.0	
% Other ethnic background	5.9	4.7	0.07
% Maternal smoking	18.0	19.1	0.74
% Paternal smoking	30.1	23.9	0.10
% Dampness in the home	19.1	16.7	0.48
% Home mould	10.1	11.2	0.68
% With ever asthma	15.1	15.1	1.00
% With current asthma	10.2	11.2	0.73
% With ever wheeze	29.3	26.8	0.52
% With current wheeze	12.4	13.4	0.72
*Among children with ever asthma*			
% With ever wheeze	92.3	89.3	0.66
% With current wheeze	44.0	58.9	0.20

## Discussion

We found a decreasing risk of asthma along an urban-rural gradient with asthma less frequent in the rural group. However, this pattern was either non-existent or less pronounced when considering other asthma-like symptoms or allergic disease. Despite the lower prevalence of asthma in rural areas, children with asthma who lived in rural areas were more likely to wheeze or have more severe symptoms of wheeze.

An increasing number of studies have investigated urban-rural differences or the farming effect in relation to asthma [1–3, 12–14]. While there has been some inconsistency in results, generally, a lower prevalence has been observed for rural or farm dwelling. Considering a gradient of urban-rural will increase the variation in exposures allowing a more informative examination of the topic. However, investigation into an urban-rural gradient in relation to asthma has been considered in a limited number of previous studies of adolescents [3, 16] and children [15, 17].

One USA study using administrative databases (birth cohort into the 6^th^ year of life) found a small but statistically significant increase in the likelihood of asthma diagnosis in rural and suburban children compared to urban children [17]. In a separate study among children from Austria (mean age 8.4 years) it appeared that the prevalence of diagnosed asthma was similar for town, rural, and farm children, with the exception of farm children who were regularly exposed to either animal sheds, hay lofts, or farm milk where asthma prevalence was lower [15]. Similar protective effects for current wheeze (wheeze in the past 12 months) were observed with regular exposure [15]. Among adolescents (aged 11–15 years), results from an earlier cross-Canada study found a dose response gradient of lessening risk of current asthma from metropolitan to non-metropolitan adjacent to rural areas [3]. These results were not seen for current wheeze [3]. A limitation of this earlier Canadian study was that the analysis was based on secondary data from a health behaviour survey that did not focus on lung disease and therefore did not consider additional confounders or lung health specific information. Results from a study in Chile (among children aged 13–14 year olds) showed that there were no statistically significant differences in asthma prevalence between urban, semi-urban, or rural dwellers while there was a significant reduction in the prevalence of current asthma symptoms (wheeze) from urban to rural dwelling [16]. This study was limited in that it only controlled for sex and current smoking status. Our current study confirms the results of the earlier Canadian study and expands on it through a more concentrated focus on lung health, a wider age range, and an investigation of the characteristics of those with asthma.

Much of the previous work investigating asthma in rural areas has focused on farming exposures. These exposures have been used to explain the previously observed lower prevalence in rural areas [29, 30]. In our study, we considered the category of rural, which included both non-farm and farm dwellers. In our previous studies within rural regions of the province, we found that the prevalence of asthma and asthma-like symptoms, as well as objective measures of atopy, were similar between farm and non-farm rural dwellers [12, 13, 31]. We hypothesize that within Saskatchewan, the rural dwelling population is relatively homogenous. To further justify our use of a rural category as opposed to splitting this variable into two groups, we compared those living in rural non-farms and rural farms and only found level of maternal education to be statistically significant between the two groups with no differences in the prevalence of asthma or wheeze.

We found that among those with asthma, there were dose–response trends of increasing prevalence of ever wheeze, current wheeze, wheeze limiting speech, wheeze with exercise, >3 episodes of wheeze in the past 12 months, and nocturnal symptoms from large urban through to rural dwelling with similar trends after adjustment for potential confounders. These results suggest that among those with asthma, dwelling in rural areas, and to a lesser degree the small urban areas, is associated with increased asthma morbidity. This may be due to higher exposure to environmental triggers or improper management and should be the focus of future investigation. It could also be due to less access to specialists to help manage asthma.

Although living in a rural area was associated with a reduced likelihood of using breathing medications in the past 12 months overall, among those with asthma, there was not a statistically significant difference between the dwelling groups. However, we did not investigate the type of medications used or their appropriateness, which could differ and lead to uncontrolled asthma and increased morbidity in the rural group. Along similar lines, among children with asthma, there were differences between location of dwelling with regard to frequency of visiting a physician for asthma. Those living outside of the large urban areas were less likely to visit a physician for asthma once in the past 12 months but not statistically different from large urban dwellers to visit a physician for asthma more than once in the past 12 months. Although we did not assess asthma severity categories in this study, we interpret this to mean that rural and small urban dwellers are less likely to visit a physician until the condition is more severe. Reasons for this could include longer travel time to health care access.

In a previous study from Arkansas, USA, comparing markers of asthma morbidity between urban and rural dwellers, it was reported that rural dwellers had an increased report of respiratory symptoms, school absenteeism, exercise limitations, and rescue medication use, despite a similar prevalence of diagnosed asthma [32]. We support the finding of increased morbidity in the rural region but the prevalence of asthma in the rural region observed in our study is lower than that in the earlier study (19% [32] vs. 15%). There were some notable differences between the two studies where the earlier study included rural regions from impoverished areas with a high prevalence of African Americans along with very different medical systems in Canada compared to the USA. In a separate study from Tennessee, USA, administrative databases were used to investigate asthma prevalence and health care utilization [17]. In this study, an urban-rural gradient was considered (urban, semi-urban, and rural). The prevalence of asthma diagnosis was higher in both non-urban areas. While the number of outpatient visits for asthma was higher in rural compared to urban areas, inhaled corticosteroid use and emergency department visits for asthma were less likely in the rural population, suggesting poor asthma control and management in the rural areas. In this earlier study, the sample sizes were quite large with small strength of associations. While we support the findings of differential physician visit practices between regions, we did not consider health care utilization in our study. Also, this earlier study used different methodology in a region of different demographic characteristics and health care systems than our study.

Reasons for the observed patterns are difficult to explain. There is a great deal of variation in personal characteristics and environmental characteristics as well as differences in access and health systems between urban and rural settings as well as between regions. In addition to this, some previous work has showed that associations may vary depending on phenotype [33, 34] and specific exposures [15]. Future work should focus on these issues as well to better disentangle these complex relationships.

We must consider limitations of our study. First, this was a cross-sectional study and comes with limitations inherent in this design including lack of temporality. However, because we were examining prevalence across regions, a cross-sectional study is an appropriate design. Second, our data was collected from proxy (parental) completed questionnaire. This can result in potential recall bias. Despite this, survey responses regarding asthma have been shown to be relatively accurate and are often the methods of choice in large epidemiological studies due to practical constraints such as cost and efficiency [35]. A limitation of our study could be that we collected data in spring when outdoor allergens and seasonal allergies are most common which could results in asthma symptoms. Despite this, if symptoms occurred due to the season, report on the questionnaire would likely be more accurate than recall in the past 12 months during a time when seasonal exposures were low and related symptomology not present. There are a great deal of potential predictors and confounders that may explain the observed associations and investigation of these relationships goes beyond the current analysis. Future work should focus on these associations. Finally, the participation rate we experienced was moderate and somewhat higher in the rural population. Because of this, we cannot exclude the possibility that there may be response bias in our sample such as more participation from those with asthma or asthma-like symptoms. While this may inflate our levels of prevalence, we would expect that this would occur non-differentially between locations allowing our interpretation of the results comparing regions to remain valid. Also, the levels of prevalence we report herein, are similar to those reported previously from the province [12, 13, 27] including one study with over 90% response rate [27]. In addition to this, our sex distribution for this age group is similar to that for each region under study (large urban: 49%; small urban: 49%; rural: 48%) [36]. Because of the higher prevalence of asthma in males in this age group [37], if there was a biased sample, we would expect a higher proportion of males taking part. We do not see this, increasing our confidence in the external validity of the results.

Our study also had several strengths. We included a large sample size from across an urban-rural gradient. This allowed us adequate statistical power and the opportunity to consider asthma and related conditions across a continuum to investigate a potential dose–response effect. Our classification of dwelling location was based on national methods of location assessment (the Beale Codes) which considers both population size, density, and distance to metropolitan areas. We used a standardized survey across all the regions included in this study, which was based on previously used validated surveys.

## Conclusions

In conclusion, we found a dose response association with lower risk of asthma among rural dwelling children. While the prevalence of asthma was lower in rural areas, it was still relatively high. As such, we emphasize the importance of focusing on asthma among rural dwellers, especially given the evidence showing that among those with asthma, living in a rural area is associated with more severe symptoms. There should be continued investigation of environmental factors in relation to asthma presence and morbidity but we must also consider other directions of research including the study of patient presenting and diagnostic labeling patterns, more in-depth analysis of which characteristics explain the observed associations, and further clinical investigation including objective lung health measures, phenotyping, and the use of biomarkers. This future research will assist in furthering the understanding of asthma etiology as well as identifying populations who require additional attention and management.

## Abbreviations

CI: Confidence interval
OR: Odds ratio
p: P-value
SPSS: Statistical Package for the Social Sciences
USA: United States of America
vs: Versus
